# Hammerstein–Wiener Multimodel Approach for Fast and Efficient Muscle Force Estimation from EMG Signals

**DOI:** 10.3390/bios12020117

**Published:** 2022-02-13

**Authors:** Ines Chihi, Lilia Sidhom, Ernest Nlandu Kamavuako

**Affiliations:** 1 Department of Engineering, Campus Kirchberg, Faculté des Sciences, des Technologies et de Médecine, Université du Luxembourg, 1359 Luxembourg, Luxembourg; 2 Laboratory of Energy Applications and Renewable Energy Efficiency (LAPER), El Manar University, Tunis 1068, Tunisia; lilia.sidhom@enib.rnu.tn; 3 Department of Engineering, King’s College London, London WC2R 2LS, UK; ernest.kamavuako@kcl.ac.uk; 4 Faculté de Médecine, Université de Kindu, Kindu, Democratic Republic of the Congo

**Keywords:** electromyography (EMG) signals, Hammerstein–Wiener model, multimodel, artificial neural network, muscle force

## Abstract

This paper develops a novel approach to characterise muscle force from electromyography (EMG) signals, which are the electric activities generated by muscles. Based on the nonlinear Hammerstein–Wiener model, the first part of this study outlines the estimation of different sub-models to mimic diverse force profiles. The second part fixes the appropriate sub-models of a multimodel library and computes the contribution of sub-models to estimate the desired force. Based on a pre-existing dataset, the obtained results show the effectiveness of the proposed approach to estimate muscle force from EMG signals with reasonable accuracy. The coefficient of determination ranges from 0.6568 to 0.9754 using the proposed method compared with a range of 0.5060 to 0.9329 using an artificial neural network (ANN), generating significantly different accuracy (*p* < 0.03). Results imply that the use of multimodel approach can improve the accuracy in proportional control of prostheses.

## 1. Introduction

Since 1952, the relationship between electromyography (EMG) and muscle force has been the focus of research for many applications ranging from prostheses control to active user-driven exoskeletons. Different approaches based on empirical and mechanical models are proposed for estimating muscle forces from the EMG signals. As the name implies, mechanical models are developed from physical laws (mechanics, biologics, electric, etc.). They are to be helpful in applications where individual muscle kinetics and kinematics are of interest. Hill’s muscle model is considered the most used physical model in musculoskeletal application to study phenomena in which only mechanical behaviour is considered [1,2,3,4]. However, this model has not shown its complete suitability in the control of upper limb myoelectric prosthesis.

Within our scope for controlling upper limb myoelectric prostheses, accurate estimation of the overall force from the EMG drive proportional control-based schemes. Therefore, many black-box models and statistical identification techniques are proposed [5,6,7,8,9,10,11]. The degree of linearity between the EMG and muscle force was debated for many years. Staudenmann et al. (2010) showed in a review paper that the relationship between EMG muscle activities and muscle force is not necessarily linear from a physiological and biophysical perspective. It depends on the level of recruitment and, therefore, on the composition of the type of muscle fibres [11,12,13,14,15,16,17]. Indeed, muscle force is modulated by the number of motor units recruited and their activation frequency [18,19], explaining the nonlinear relationship between EMG and muscle force [20,21]. In [22], experiments based on a recruitment range controlled by an electrical stimulation protocol showed that the EMG–muscle force relationship could be assimilated as a linear relation for muscles constituted with one type of fibre. However, mixed-fibre-type muscles, composed of fast and slow switching fibres, exhibit a nonlinear relationship between muscle activities and forces. Furthermore, Kamavuako and Rosenvang (2012) provided evidence of hysteresis in the EMG–force relationship to indicate non-linearity [23].

From a mathematical modelling point of view, several estimation methods [24,25,26,27,28,29,30] were proposed in the literature, including linear and nonlinear approaches with different outcomes. In this perspective, Hahne et al. proposed a comparative study of linear and nonlinear regression techniques to estimate muscle force from the EMG signals for myoelectric control movements. This study was based on four estimation algorithms: linear regression (LR), mixture of linear experts (ME), multilayer perceptron (MLPs), and Kernel ridge regression (KRR). They showed that, on the whole, the interest of nonlinear methods, especially the KRR method, is considered a nonparametric and nonlinear statistical learning method [27]. Luo et al. (2019) [30] proposed a three domains fuzzy wavelet neural network algorithm without prior knowledge of the biomechanical model to analyse the force muscles. This approach is based on the mean absolute value of the electromyographic signal, used as the input for the potential model. Recently, Wimalasena et al. adapted a new AutoLFADS approach based on unsupervised deep learning, more precisely, recurrent neural networks (RNNs). This approach is applied to estimate muscle activities from multi EMG signals [31].

The superiority of nonlinear techniques supports the non-linearity of the EMG–force relationship, especially at higher contraction levels when many motor units are recruited. Non-conventional black-box models, such as artificial neural network-based methods, machine learning, or deep learning approaches, are suggested for modelling complex systems. These approaches are proven effective in predicting force, especially for nonlinear systems; nevertheless, they are considered complex techniques in modelling architecture and training methods [32,33,34]. Despite this, many non-conventional techniques only show good performance in specific tasks due to “Catastrophic Forgetting”, which is the problem of losing information on a first task, T1, after training a second task, T2. Otherwise, the performance of T1 will significantly decline [35,36,37,38]. Additionally, non-conventional techniques are costly for production use. Indeed, complex problems require an extensive network that can be exceptionally time-consuming to compute at inference times [5,6,7,8]. To address the challenges encountered in modelling the non-linearity of the EMG–force relationship for upper limb prostheses, and thus to overcome the disadvantages of the techniques above, in this study, we propose a multimodel approach. A multimodel approach is suggested for nonlinear system modelling to decompose a whole operation area of a studied process into a defined number of sub-operating regions. In each one, a local model is computed. This approach is successfully applied even when dynamical system characteristics vary considerably over the operating regime. Thus, the multimodel concept is considered an exciting method to improve the model’s performance in terms of precision and without overly increasing the computational burden or the number of parameters to adjust [39,40,41,42,43,44].

## 2. Related Studies

We propose a novel MIMO multimodel structure to model muscle force from the EMG signals in the present study. Sub-models of the proposed design are based on the nonlinear Hammerstein–Wiener (H–W) model, which was applied in different fields to estimate complex and nonlinear systems [45,46,47,48,49,50,51,52,53,54,55]. Indeed, the H–W model was used in some works to estimate muscle force from EMG signals. In this context, Kumar et al. (2011) proposed a nonlinear modelling approach to characterise the relationship between EMG and muscle force in [47]. Their study developed a hybrid model based on the fusion technique between multi nonlinear autoregressive exogenous (ARX) and Hammerstein–Wiener (H–W) models designed for different force/EMG data. Each force sensor model’s output is first fused using an adaptive probability algorithm. Secondly, the outputs of various sensors are combined by the same adaptive algorithm to estimate the desired force. Despite the acceptable results, the proposed approach is significantly complex because of the high number of nonlinear models and parameters to be calculated for each hybrid model developed for each force sensor.

In 2011, a H–W based model was proposed to estimate one muscle force from one EMG Signal. The proposed approach shows that despite the acceptable error rate shown in the direct validation of the H–W model, a significant error is obtained in cross-validation. This approach delivers good performance only for particular data [48]. Sebastian et al. (2011) presented an analysis of different filtering methods to measure the dynamics of surface EMG signals. This analysis was compared based on the EMG finger force model based on H–W estimation to show that, in this case, the nonlinear spatial filters gave better-fit values [50]. Indeed, regardless of the application, approaches based on the H–W model show significant performance to mimic nonlinear systems [48,49,50,51,52,53,54,55]. However, this technique requires parameter adjustment for each new dataset. Furthermore, the real and the estimated force error is significant, especially in cross-validation [39,40,41]. This uncertainty can affect the quality of monitoring or control. Additionally, most of the proposed models characterise just one force profile/channel from EMG signals. However, it is most interesting for complex movements to simultaneously model two or more muscle forces. In this work, based on a multimodel approach, we propose a MIMO muscle force estimator from EMG signals of the upper limb using diverse force profiles. The novelty of the proposed approach lies in (1) its efficiency in estimating force with a minimal number of parameters; (2) ability to optimise the number of used EMG signals to estimate multiple muscle force profiles; (3) stable performance with direct and cross-validation with a minimal computational burden. To show the efficiency of the proposed modelling structure, we have compared it with the artificial neural network (ANN) modelling technique known for its effectiveness, especially for data sets having nonlinear relations [27,56].

The paper is organised such that, in Section 3, we describe the proposed multimodel structure of the proposed experimental approach and define a sub-section for data analysis. The validation of the developed method and a comparative study with the ANN model are presented and interpreted in Section 4. Section 5 discusses and interprets the results from different scenarios that we developed to validate the multimodel approach. Finally, the conclusion and perspective are presented in Section 5.

## 3. Methods

**Figure 1:**
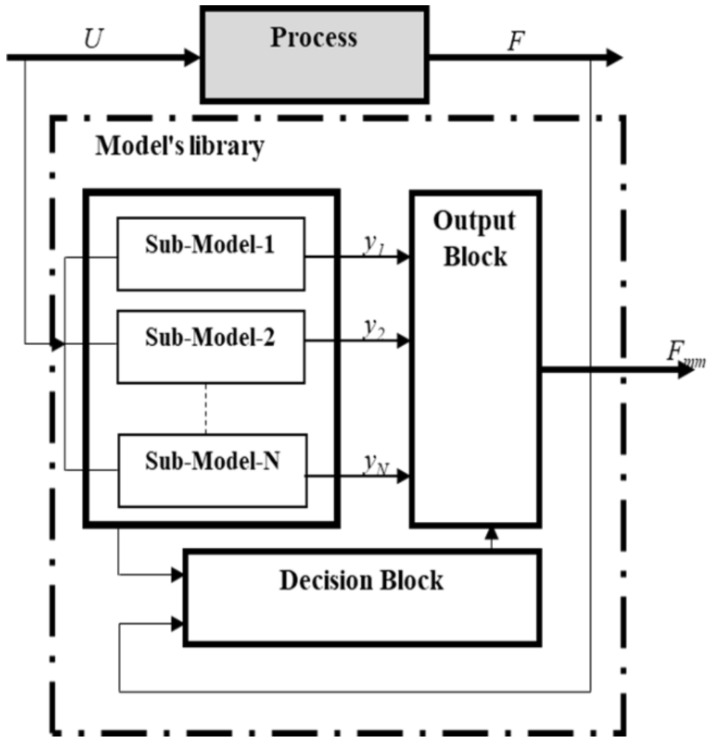
Magnetisation principal of multimodel approach.

The multimodel technique consists of replacing a unique representation of a complex dynamic system with a nonlinear regime by combining multiple models, called sub-models. Different measurement pairs developed each sub-model to describe the behaviour of the studied system in its whole operating domain at a particular operating point. Figure 1 show the principle of system modelling using a multimodel approach. Sub-models are grouped into a model base, named library. We note that they can have different structures or/and orders; however, no one can describe the studied nonlinear process in its whole operating area. Based on the interaction of the sub-models, the decision unit allows us to estimate the contribution of each sub-model in a weighted manner according to their posterior likelihoods to foster the most pertinent one at each time. The decision block controls the output block to compute the output of the multimodel structure, Y mm, which is obtained by the fusion or the switching of the different sub-model responses.

To explain and simplify the basic principle of the proposed MIMO multimodel structure, we suggest, for example, fixing the number of inputs and outputs to 2, i.e., we have to estimate two muscle forces, *F_m1_* and *F_m2_*, from two EMGs signals, *EMGm1* and *EMGm2*.

**Figure 2:**
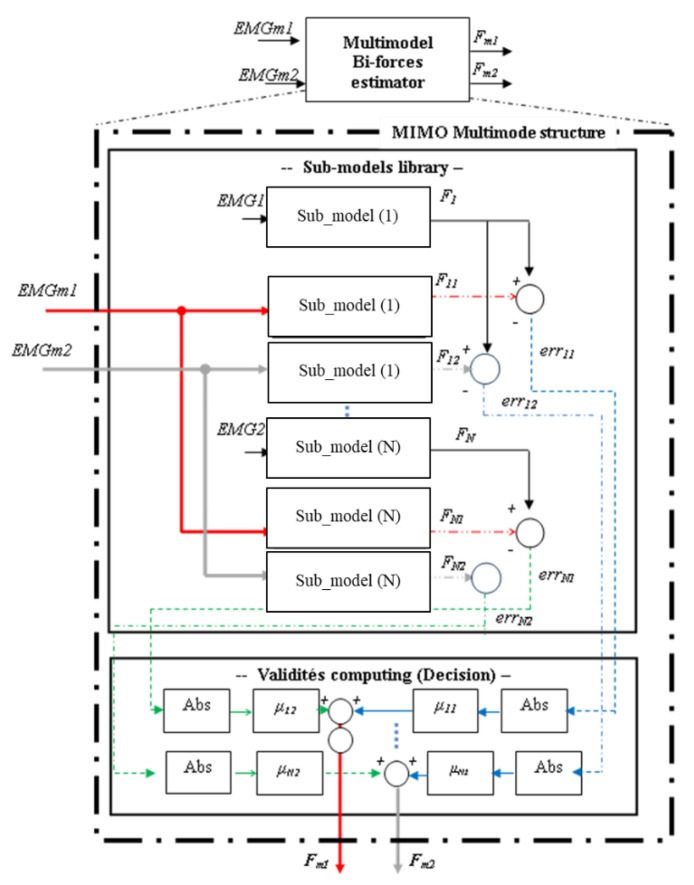
Multimodel structure to estimate two muscle forces *F_m1_* and *F_m2_* from two EMG signals *EMGm1* and *EMGm2*.

The proposed multimodel structure is described by the following steps (the different steps will be detailed in the next section), as shown in Figure 2:

For Figure 2, we define:
*EMGm1* and *EMGm2*:Inputs of the multimodel bi-forces estimator.*F_m1_* and *F_m2_*:Outputs of the multimodel bi-forces estimator.*EMGN*:Input of sub-model (i). *F_i_*:Output of sub-model (i).*F_i1_* and *F_i2_*:Output of sub-model (i) obtained by applying multimodel inputs, *EMGm1* and *EMGm2*, respectively.*err_i1_* and *err_i2_*:Errors of sub-model (i) computed between its real output, *F_i_*, and outputs, *F_i1_* and *F_i2_**μ_i1_* and *μ_i2_*:Validities of sub-model (i) according to *F_m1_* and *F_m2_*, respectively.

- **Step 1_Elaboration of sub-models**: Definition of sub-models of the library: Sub-model (i): model defined for the measurements couple (*EMGi*, *F_i_*).
- **Step 2_Normalised residues estimation**: Computation of the normalised error of each sub-model (i): *err’_i1_* and *err’_i2_*.
- **Step 3_Validity Computing**: Computation of the weight, also named validity, of each sub-model: *μ_i1_* and *μ_i2._*
- **Step 4_Outputs Computing**: Computation of outputs *F_m1_* and *F_m2_*.

*i = 1, 2, …, N*, with *N* is the total number of sub-models.

Below is the explanation of the four steps.

**Step 1_Elaboration of sub-models:** In terms of duration, form, and even complexity, the considered experimental data present different force profiles. Furthermore, the complex and unpredictable characteristics of EMG signals and the anatomical and physiological properties of muscles justify the choice of the Hammerstein–Wiener (H–W) structure to estimate muscle force. Indeed, this structure considers inputs and outputs as nonlinear information [51,52,53,54,55]. It allows observing input/output measurements to develop a model that approximates the behaviour of the underlying system. Furthermore, a nonlinear H–W model, considered a black-box method, can be presented as a concatenation mapping between previously observed data and a regression space followed by a nonlinear function. In addition, in our case of study, the data analysis stage, detailed in section B, shows the non-linearity of the inputs and outputs, promoting the use of the H–W regressor and justifies our choice to utilise it for this study.

The following equations can formulate the system identification approach:

(1) U=[U(1), U(2), …, U(kt)]

(2) F=[F(1), F(2), …, F(kt)]

The following general dynamic model can also represent the Output vector:

(3) $$F=g(u^{k-1},\;f^{k-1},\;\theta \left(k\right)+v\left(k\right)$$

where:

*v(k):* Noise signal, presenting the prediction of error between actual and estimated output.

In the case of an ideal model and good prediction of *F, v(kt)* approaches zero, and the mapping function is as follows:

(4) $$g\left(u^{k-1},\;f^{k-1},\;\theta \left(k\right)\right)=g\left(\phi \left(k\right),\;\theta \left(k\right)\right)$$

with:



*ɸ(k)*
: Observation vector, also named regression vector, contains inputs and outputs data of previous instants. *ɸ(k)= ɸ(u^k–1^ , f^k–1^)*
*θ(k)*
: Parameter vector *θ = [θ_1_, θ_2_, …, θ_p_] p* is the number of parameters to be estimated.


The parameters of the HW models are calculated during the training phase based on the parametric identification algorithm recursive least squares algorithm (RLS). The evolution of these parameters shows that they converge towards a constant value, making it possible to consider fixed parameters that do not need readjustment. The following equations can describe the RLS algorithm:

(5) $$\overset{\wedge }{\theta }\left(k\right)\;=\overset{\wedge }{\theta }\left(k-1\right)\;+\;P\left(k\right)\;\overset{k}{\underset{i=n+1}{\sum }}y\left(i\right)\;\phi \left(i\right)$$

(6) $$P\left(k\right)=\;P\left(k-1\right)\;-\;\frac{P\left(k-1\right)\;\phi \left(k\right)\;\phi^{T}\left(k\right)\;P\left(k-1\right)}{1\;+\;\phi \left(k\right)\;P\left(k-1\right)\;\phi \left(k\right)}$$

(7) $$\varepsilon \left(kt\right)\;=\;y\left(kt\right)\;-\;\overset{\wedge }{\theta }\left(kt-1\right)\;\phi \left(kt\right)$$

where:

$\overset{\wedge }{\theta }$ (*k*): vector of estimated parameters,

*P* (*k*): adaptation matrix,

ε (*k*): estimated error,

*y* (*k*): actual output of the system to identify,

*k:* is the discrete-time.

**Figure 3:**
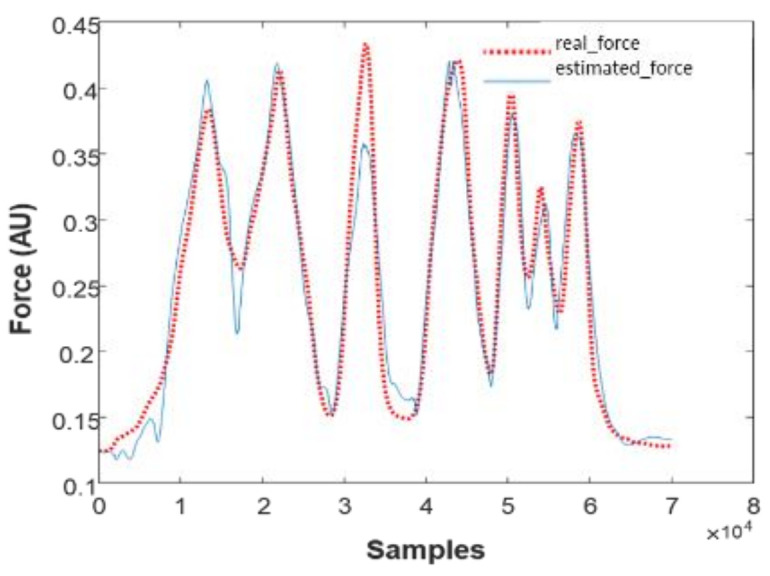
Responses of sub-models based on Hammerstein–Weiner structure.

The sub-model given above was implemented, and the result is summarised in Figure 3 for a representative subject.

**Figure 4:**
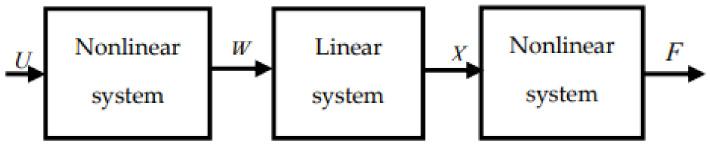
The general structure of the Hammerstein–Weiner model.

We can notice a good correlation between the real muscle force and the response of the model. Parameters estimation of the H–W sub-models is based on the recursive least squares algorithm [57,58]. We note that the actual force is a continuous blue line and the estimated force is the dotted red line. We note that the response of the developed H–W sub-models is similar to the experimental data. However, these models are limited if we change input/output data by applying experimental data from which one sub-model was developed to another sub-model referring to the same type of force profile. In this sense, the limit of H–W models will be overcome with the multimodel approach. As shown in Figure 4, Hammerstein–Wiener can be presented by a serial association of nonlinear, linear, and other nonlinear systems.

**Step 2_Normalised residues estimation:** In the literature, different approaches were proposed to estimate validity degrees of sub-models [41,59,60] that can be defined by a switching function or by fusion of sub-model responses. In our study, we use a fusion technique based on computing normalised residues, *err’_i1_* and *err’_i2_*, of each sub-model to evaluate its pertinence to describe the system in an operating area at each time, estimation of errors between the real and the estimated outputs:

(8) $$\begin{array}{l} err_{i1}(k)=\left|F_{i1}(k)-F_{1}(k)\right|\\ err^{\prime }{}_{i1}(k)=\frac{err_{i1}(k)}{\sum_{i=1}^{N}err_{i1}(k)} \end{array}\begin{array}{l} \;err_{i2}(k)=\left|F_{i2}(k)-F_{2}(k)\right|\\ \;err^{\prime }{}_{i2}(k)=\frac{err_{i2}(k)}{\sum_{i=1}^{N}err_{i2}(k)} \end{array}$$

**Step 3_Validity Computing:** Validity coefficients must belong to the interval [0,1] and vary contrary to residual values [42,43,44,45]. Otherwise, the most pertinent model must have the minor residue and the most important validity value.

(9) $$\begin{array}{l} \mu_{1i}(k)=1-err^{\prime }{}_{i1}(k)\\ \mu_{2i}(k)=1-err^{\prime }{}_{i2}(k) \end{array}$$

*μ_1i_* and *μ_2i_* are designed validities of sub-model (i) according to *F_m1_* and *F_m2_*, respectively.

**Step 4_Outputs Computing:** The multimodel outputs *F_m1_* and *F_m2_* are computed as follows:

(10) $$\begin{array}{l} F_{m1}(k)=\sum_{i=1}^{N}\mu_{1i}(k)F_{i}(k)\\ F_{m2}(k)=\sum_{i=1}^{N}\mu_{2i}(k)F_{i}(k) \end{array}$$

### 3.1. Experimental Approach

**Figure 5:**
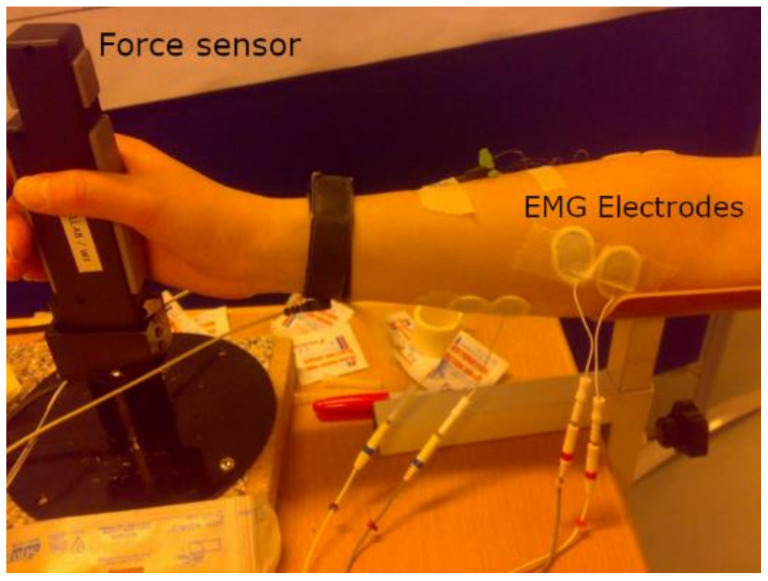
The considered experimental approach.

The proposed study makes use of pre-existing data [56]. In brief, 10 able-bodied human subjects (7 w/3 m; age range, 21–37 years, mean 26.9 years) took part in this experiment following the Declaration of Helsinki (approval no.: N-20080045). Each subject received an information sheet about the investigation and gave written consent before participation. Subjects were asked to follow six force profiles randomly assigned. The six force profiles were defined as follows: (a) a step increase in static grip force with five increments of a 10 s duration of 10N (step), (b) two linear ramps (saw), (c) a freely varying force (vol), and (d) a steady-state force at 50N (single-level); a slow increase and decrease for a duration of 10 s (circle). Four trials were recorded for each force profile, and a rest of 1 min followed each practice. In total, 160 trials were collected. The force produced during power grip was measured using a commercially available handgrip dynamometer (Vernier Software & Technology, accuracy ± 0.6 N, operational range 0–600 N, grip size 50 mm × 25 mm). Surface EMG was measured in a bipolar configuration using disposable Ag/AgCl surface electrodes (Ambu Neuroline 720, Denmark) from the M. extensor carpi radialis (ECR), flexor digitorum superficialis (FDS), and flexor carpi radialis (FCR). The sEMG signals were amplified by a factor 2000 with a multichannel surface EMG amplifier (EMG16, LISiN, Torino, Italy), and band-pass filtered (20–500 Hz) and sampled at 1000 Hz. Figure 5 present the experimental setup to measure the force profile and EMG signals from the forearm muscle.

### 3.2. Data Analysis

A data processing step was applied to digitally rectify the force and EMG signals before the estimation stage. We have recovered the envelope of the signals with a low pass filter using a 6th order Butterworth filter to cut off frequency at 1Hz and finally normalise the data accordingly using Min–Max normalisation in the data preprocessing stage. The Min–Max normalisation is defined as follows:

(11) $$d_{n}=\frac{d_{r}-d_{\min}}{d_{\max}\;-\;\;d_{\min}}$$

with:

*d_n_*: the normalised value,

*d_r_*: the real value,

*d_min_*: the minimum value,

*d_max_*: the maximum value.

Then, data were sent to the proposed approach, as described above, for force estimation and compared with performance based on the artificial neural network as previously suggested. The appropriate number of sub-models depends on the different operating domains that can describe the whole behaviour of the studied nonlinear process. It is fixed according to statistical analysis of the EMG data. Indeed, the multimode approach is generally proposed to optimise the characterisation of a nonlinear system having different operating points. A sub-model represents each operating point. However, in this study, we have input/output type models (black-box models) possessing the same structure but different non-physical parameters that change according to the change of the data, especially the EMG inputs, considered unpredictable signals and challenging to characterise.

**Figure 6:**
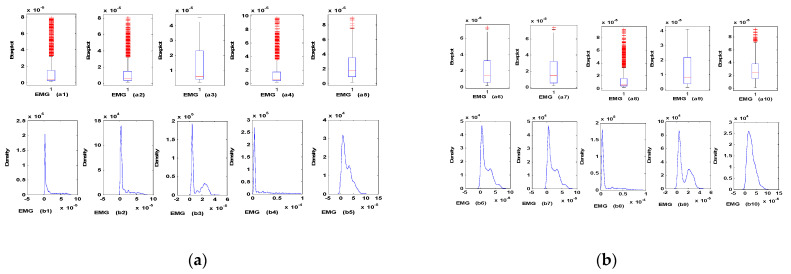
Data analysis (box plots and density profiles) of EMG signals for the movement “Two linear ramps.” (**a**) Box plot of the EMG signal *i* (**b**) density of EMG signal *i*.

Furthermore, EMG is asynchronous and can be assimilated to a succession of irregular and unpredictable wave trains. Therefore, we analysed the recorded data of EMG signals relating to each type of movement to study and determine the number of data classes in terms of distribution and statistical characteristics. The number of sub-models N is then relative to these classes. Otherwise, each sub-model has a well-determined distribution class of EMG inputs. Figure 6 represent the box plots and the distributions of the EMG signals, measured for the same movement "Two linear ramps (saw)." We remark that we have mainly three distinct behaviours of these data, which allows us to propose three sub-models; each presents a different statistical characteristic.

Based on this consideration and to show the ability of the proposed multimodel approach to estimate different kinds of force profiles (FP), we present the following scenarios:

- **Scenario1:** Predict force profiles from sub-models characterising the same kind of desired forces. For example, predicting a step from another instance of the step profile.
- **Scenario2:** Predict profiles of arbitrary forces from sub-models characterising 02 known forces. For example, using step and circle to estimate free profiles (vol).
- **Scenario3:** Predict profiles of arbitrary forces from sub-models characterising 03 known forces. This is the same as scenario 2, but with three standard profiles. For the different scenarios, we note that inputs/outputs of the multimodel approach are considered unknown, and any sub-model of the library does not represent them. Three performance measures: coefficient of determination (R2), root mean squared error (RMSE), and computational time (CT), were used to assess the proposed approach. For each performance metric, a two-way repeated measure analysis of variance (ANOVA, IBM SPSS Statistics 26) with factors methods (multimodel vs. ANN) and scenarios (1,2 and 3) was used to assess the performance of the proposed approach. Table 1 present the computational environment used in this paper.

**Figure 7:**
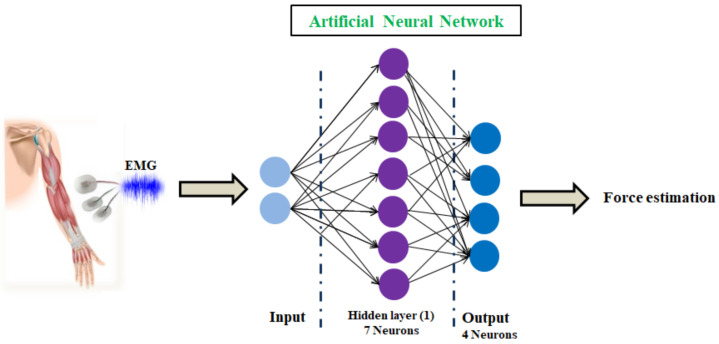
The architecture of the applied ANN algorithm.

The architecture of the considered ANN algorithm can be described as follows, Figure 7:


Number of feed-forward network layers: 2Number of hidden layers:1Number of neurons in the hidden layer:7Type of activation function:Tangent SigmoidBatch training method:Levenberg–MarquardtNumber of output neurons:4


## 4. Results

**Figure 8:**
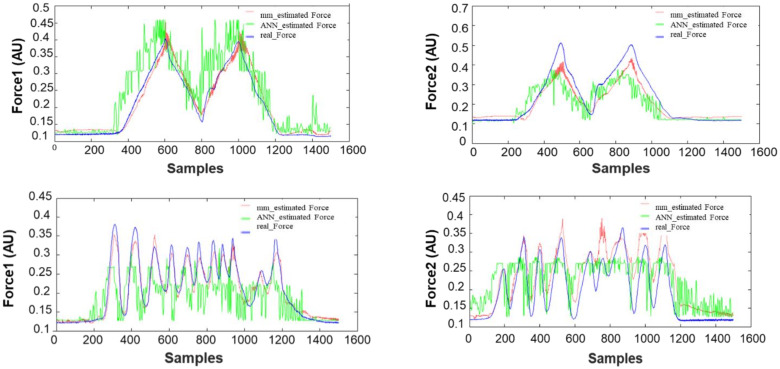
The Representative performance of the multimodel approach compared with ANN.

Figure 8 show the ability of the developed approach to predict different kinds of force profiles from surface EMG signals. This figure illustrates the simulation results of the proposed scenarios to show the interest of the proposed approach compared with the artificial neural network in the prediction of forces. CT was 110.1 ± 13.4 ms and the multimodel approach was significantly lower (*p* < 0.01) than the ANN (825.0 ± 136.0 ms). Indeed, the fast training of the proposed method allows an online implementation to deal with continuous data preprocessing and generate more efficient real-time estimation. The overall results of the scenarios are summarised in Table 2 for R^2^ and RMSE, respectively. For R^2^, the proposed approach (0.876 ± 0.045) outperformed (*p* < 0.001) the ANN (0.738 ± 0.066). There was a significant difference between scenarios, with scenario 3 performing worst (*p* = 0.24). Furthermore, Table 1 show a significant interaction (*p* = 0.028) between approaches and scenarios. The overall results of the scenarios are summarised in Table 1 and Table 2 for R^2^ and RMSE, respectively. For R^2^, the proposed approach (0.876 ± 0.045) outperformed (*p* < 0.001) the ANN (0.738 ± 0.066). There was a significant difference between scenarios, with scenario 3 performing worst (*p* = 0.24). Furthermore, Table 2 showed a significant interaction (*p* = 0.028) between approaches and scenarios. The RMSE of the proposed approach was 0.026 ± 0.006, significantly (*p* < 0.001) lower than ANN (0.039 ± 0.009). RMSE was different (*p* = 0.021) between scenarios but there was no interaction (*p* = 0.068), Table 3.

**Figure 9:**
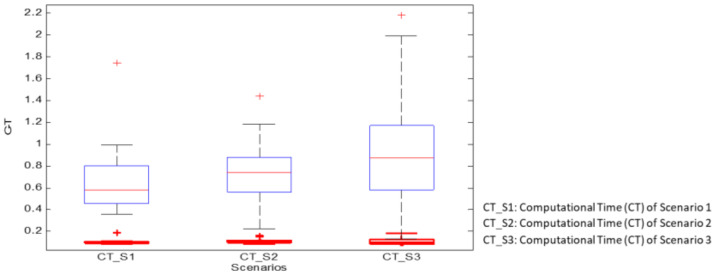
Performance (for ten subjects) of force estimation evaluated with training computing time for three scenarios (multimode approach is represented by the red line and ANN the blue one).

Table 4 and Figure 9 show a comparative study between the multimode approach and ANN regarding computation time measured for the different subjects according to the proposed three scenarios. This study shows the interest in the multimode approach compared to the ANN. Indeed, the computation time of the mm approach is 10 to 20 times less than that of the ANN method. It is also almost constant for the different scenarios, unlike the ANN approach, where the computation time depends on the scenario. In addition to the computational time, the memory requirements are also important to consider. In this perspective, Table 5 present the memory requirements for both methods using the computational environment described in Table 1. Indeed, the ANN approach requires almost 25% more memory than the multimodel approach.

## 5. Discussion

This study proposed a multimodel approach based on nonlinear Hammerstein–Wiener to estimate force from EMG with the MIMO feature that enables multiple force profiles to be learned simultaneously as sub-models. The concept of force estimation from EMG is essential for the proportional control of myoelectric prostheses [2,3,4]. Overall, the proposed approach outperformed the artificial neural network for all metrics used in the study. The architecture of the ANN used in the study is the same that was used by [47] to allow direct comparison. We have deliberately chosen not to compare with other H–W models because our target application is myoelectric control.

Furthermore, ANNs are established as the golden standard for force estimation due to the poor performance of linear regression, especially for simultaneous force estimation [30,47]. By comparing a previous architecture using the same dataset, the actual performance of the proposed approach can be demonstrated. Regardless of the method used for the estimation, we see that they perform very well for simple problems (scenario 1) with a decrease in performance when the problem becomes more complex. Nevertheless, the proposed approach is more robust to changes in complexity as proven using both R^2^ and RMSE. Furthermore, the proposed method requires less computational power and time compared with the ANN. Particular attention can be brought to scenario three, where it is shown that the increase in the number of sub-models does not automatically generate an improvement in force estimation. Indeed, the choice of the type of sub-models that constitute the library of the multimodel structure is critical.

Nevertheless, the multimodel approach allows us to not only characterise arbitrary forces from EMG signals but also improve the prediction’s statistical performance, the linear resemblance between the real and the estimated force, and provide multi forces from a simple, well-defined model sub-models library. Despite the improved performance, the dataset used in the study deals with sequential force profiles, while the challenging problem with myoelectric prostheses is the ability to control motions simultaneously. This study intended to pave the way for using the approach as we are designing a novel experimental protocol that will include both offline and real-time estimation of simultaneous force profiles. However, the situation with the COVID-19 pandemic is impeding the recruitment of subjects, especially participants with limb amputations.

## 6. Conclusions

This paper aims to characterise muscle forces from electromyography signals of the upper limb. A multimodel approach was developed to extend and increase the flexibility to model several kinds of force profiles to overcome this challenge. The proposed method is mainly based on nonlinear Hammerstein–Wiener models used to reconstruct the library of the multimodel structure. The validity of each H–W sub-model is computed to estimate the contribution of each one to estimate the desired outputs. The multimodel approach was validated for different scenarios to propose libraries with varying numbers of sub-models representing the same or other types of the desired force profile. The proposed method shows acceptable metrics values in R^2^, RMSE, and computed total CPU time. However, it would be of great interest to continue the real-time experimental investigations to assess the method, especially for amputee subjects. In addition, the results of the multimodel approach are promising in terms of efficiency, computation time, and memory requirements.

## Figures and Tables

**Table 1:** Computational environment.

Experimental Environment	Proprieties
Operating system	Windows 10 Professionnel
Processor	Intel(R) Core(TM) i7-8565U CPU @ 1.80 GHz 1.99 GHz
Processor generation	8th Gen
Installed RAM	8.00 Go, (7.88 Go usable)
System Type	64 bits operating system, x64-based process
Graphics card	NVIDIA GeForce MX110
Software	Matlab 2017

**Table 2:** Evaluation of the performance in terms of R^2^.

	Scenario-1		Scenario-2		Scenario-3	
	mm	ANN	mm	ANN	mm	ANN
**Sub-1**	0.9592	0.6374	0.9742	0.7479	0.7922	0.5060
**Sub-2**	0.8923	0.8443	0.8979	0.8987	0.6568	0.5625
**Sub-3**	0.8878	0.8620	0.9466	0.9329	0.7997	0.5780
**Sub-4**	0.9754	0.8258	0.8299	0.8855	0.6015	0.6054
**Sub-5**	0.9330	0.7038	0.8867	0.8452	0.8517	0.6014
**Sub-6**	0.8963	0.8919	0.9038	0.8619	0.9090	0.6830
**Sub-7**	0.8285	0.5417	0.8087	0.8317	0.8972	0.5419
**Sub-8**	0.9584	0.8092	0.9684	0.8858	0.9062	0.8354
**Sub-9**	0.9539	0.7776	0.9033	0.5931	0.8454	0.7360
**Sub-10**	0.9326	0.6403	0.8026	0.8326	0.8912	0.6434
**mean**	**0.8927**	**0.7127**	**0.8427**	**0.6485**	**0.7559**	**0.3810**
**Max**	**0.9754**	**0.8970**	**0.9742**	**0.9329**	**0.9090**	**0.8354**
**STD**	**0.0043**	**0.0180**	**0.0099**	**0.0884**	**0.0210**	**0.1480**

**Table 3:** Evaluation of the performance in terms of RMSE.

	Scenario-1		Scenario-2		Scenario-3	
	mm	ANN	mm	ANN	mm	ANN
**Sub-1**	0.0186	0.0554	0.0201	0.0190	0.0353	0.0671
**Sub-2**	0.0348	0.0348	0.0123	0.0215	0.0471	0.0434
**Sub-3**	0.0211	0.0227	0.0230	0.0255	0.0232	0.0360
**Sub-4**	0.0173	0.0460	0.0352	0.0288	0.0477	0.0658
**Sub-5**	0.0249	0.0524	0.0217	0.0389	0.0261	0.3050
**Sub-6**	0.0300	0.0249	0.0146	0.0194	0.0193	0.0323
**Sub-7**	0.0242	0.0395	0.0227	0.0213	0.0207	0.0437
**Sub-8**	0.0167	0.0358	0.0244	0.0465	0.0275	0.0383
**Sub-9**	0.0210	0.0481	0.0107	0.0219	0.0356	0.0440
**Sub-10**	0.0261	0.0602	0.0549	0.0508	0.0308	0.0557
**mean**	**0.0304**	**0.0498**	**0.0318**	**0.0387**	**0.0396**	**0.0868**
**min**	0.0167	0.0227	0.0107	0.0190	0.0193	0.0323
**STD**	**1.7450 e-04**	**2.2470 e-04**	**0.0003**	**0.00035**	**0.00025**	**0.0091**

**Table 4:** Scenarios 1 to 4: evaluation of obtained results for ten subjects in terms of training computation (10^2^ s).

	Scenario-1		Scenario-2		Scenario-3	
	**mm**	**ANN**	**mm**	**ANN**	**mm**	**ANN**
**Sub-1**	0.1114	0.3606	0.0859	0.2210	0.0859	0.2210
**Sub-2**	0.1014	0.6231	0.1191	0.8610	0.0977	1.1734
**Sub-3**	0.0989	0.8061	0.0986	0.6714	0.0884	0.8842
**Sub-4**	0.0911	0.5401	0.1067	0.7296	0.1245	0.8619
**Sub-5**	0.0872	0.4602	0.1155	0.4712	0.0839	0.2351
**Sub-6**	0.0952	0.4128	0.1061	0.5638	0.1028	0.5850
**Sub-7**	0.0936	1.7428	0.0930	0.8783	0.1219	1.9918
**Sub-8**	0.1006	0.9940	0.1589	1.1822	0.1477	2.1794
**Sub-9**	0.1858	0.5114	0.1545	1.4397	0.1822	0.9611
**Sub-10**	0.1089	0.7438	0.1041	0.7531	0.0833	0.7904
**Mean**	**0.1074**	**0.7195**	**0.1142**	**0.7771**	**0.1118**	**0.9783**
**Max**	**0.1858**	**1.7428**	**0.1589**	**1.1822**	**0.1822**	**2.1794**
**Min**	**0.0872**	**0.3606**	**0.0859**	**0.2210**	**0.0833**	**0.2210**

**Table 5:** Evaluation of the performance in terms of memory requirements.

	mm	ANN
**Maximum Possible Array Bytes**	2.8949 × 10^9^	3.3673 × 10^9^
**Memory Available All Arrays**	2.8949 × 10^9^	3.3673 × 10^9^
**Memory Used MATLAB**	3.9115 × 10^9^	4.9214 × 10^9^

## Data Availability

Data sharing is available if requested.

## References

[1] Allen C., Karam K.Z., Le Cam P., Hill M., Tindle T. Application of virtual reality devices to the quantitative assessment of manual assembly forces in a factory environment. Proceedings of the IECON ‘95—21st Annual Conference on IEEE Industrial Electronics.

[2] Hill A. (1938). The heat of shortening and dynamic constants of muscle. Proc. R. Soc. Lond. Ser. B Biol. Sci..

[3] Delp S.L., Anderson F.C., Arnold A.S., Loan P., Habib A., John C.T., Guendelman E., Thelen D.G. (2007). OpenSim: Open-source software to create and analyse dynamic simulations of movement. IEEE Trans. Biomed. Eng..

[4] Lai A., Schache A.G., Lin Y.C., Pandy M.G. (2014). Tendon elastic strain energy in the human ankle plantar-flexors and its role with increased running speed. J. Exp. Biol..

[5] Lee S.S.M., Arnold A.S., Miara M.D.B., Biewener A.A., Wakeling J.M. (2013). Accuracy of gastrocnemius muscles forces in walking and running goats predicted by one-element and two-element Hill-type models. J. Biomech..

[6] Ben Messaoud A., Talmoudi Ben Aoun S., Lahmari Ksouri M. (2017). A New Strategy of Validities’ Computation for Multimodel Approach: Experimental Validation. Int. J. Adv. Comput. Sci. Appl..

[7] Duncombe J.U. (1959). Infrared navigation—Part I: An assessment of feasibility. IEEE Trans. Electron Devices.

[8] Lippold O.C.J. (1952). The relation between integrated action potentials in a human muscle and its isometric tension. J. Physiol..

[9] Lloyd D.G., Besier T.F. (2003). An EMG-driven musculoskeletal model to estimate muscle forces and knee joint moments in vivo. J. Biomech..

[10] Rodriguez Martinez J., Mannini A., Clemente F., Sabatini A.M., Cipriani C. (2020). Grasp force estimation from the transient EMG using high-density surface recordings. J. Neural Eng..

[11] Wang N., Lao K., Zhang X., Lin J., Zhang X. (2018). The recognition of grasping force using LDA. Biomed. Signal Process. Control.

[12] Baldacchino T., Jacobs W.R., Anderson S.R., Worden K., Rowson J. (2018). Simultaneous force regression and movement classification of fingers via surface EMG within a unified Bayesian Framework. Front. Bioeng. Biotechnol..

[13] Staudenmann D., Roeleveld K., Stegeman D.F., Van Dieën J.H. (2010). Methodological aspects of SEMG recordings for force estimation—A tutorial and review. J. Electromyogr. Kinesiol..

[14] Alkner B.A., Tesch P.A., Berg H.E. (2000). Quadriceps EMG/force relationship in knee extension and leg press. Med. Sci. Sports Exerc..

[15] De Luca C.J. (1997). The use of surface electromyography in biomechanics. J. Appl. Biomech..

[16] Komi P.V., Buskirk E.R. (1970). Reproducibility of electromyographic measurements with inserted wire electrodes and surface electrodes. Electromyography.

[17] Potvin J.R., Brown S.H. (2004). Less is more: High pass filtering, to remove up to 99% of the surface EMG signal power, improves EMG-based biceps brachii muscle force estimates. J. Electromyogr. Kinesiol..

[18] Solomonow M., Baratta R., Shoji H., Ambrosia R.D. (1986). The myoelectric signal of electrically stimulated muscle during recruitment: An inherent feedback parameter for a closed-loop control scheme. IEEE Trans. Biomed. Eng..

[19] Vink P., Van Der Velde E.A., Verbout A.J. (1987). A functional subdivision of the lumbar extensor musculature. Recruitment patterns and force-RA-EMG relationships under isometric conditions. Electromyogr. Clin. Neurophysiol..

[20] Milner-Brown H.S., Stein R.B., Yemm R. (1973). The orderly recruitment of human motor units during voluntary isometric contractions. J. Physiol..

[21] Kukulka C.G., Clamann H.P. (1981). Comparison of the recruitment and discharge properties of motor units in human brachial biceps and adductor pollicis during isometric contractions. Medicine.

[22] Woods J.J., Bigland-Ritchie B. (1983). Linear and nonlinear surface EMG/force relationships in human muscles. Am. J. Phys. Med. Rehabil..

[23] Kamavuako E.N., Rosenvang J.C. (2012). Hysteresis in the electromyography-force relationship: Toward an optimal model for the estimation of force. Muscle Nerve.

[24] Calvert T.W., Chapman A.E. (1977). The relationship between the surface EMG and force transients in muscle: Simulation and experimental studies. Proc. IEEE.

[25] Bottomley H. (1965). Myo-electric control of powered prostheses. J. Bone Joint Surg. Br..

[26] Kamavuako E.N., Scheme E.J., Englehart K.B. (2013). Wrist torque estimation during simultaneous and continuously changing movements: Surface versus untargeted intramuscular EMG. J. Neurophysiol..

[27] Hahne J.M., Bießmann F., Jiang N., Rehbaum H., Farina D., Meinecke F.C., Müller K.-R., Parra L.C. (2014). Linear and Nonlinear Regression Techniques for Simultaneous and Proportional Myoelectric Control. IEEE Trans. Neural Syst. Rehabil. Eng..

[28] Farina D., Jiang N., Rehbaum H., Holobar A., Graimann B., Dietl H., Aszmann O.C. (2014). The extraction of neural information from the surface EMG for the control of upper-limb prostheses: Emerging avenues and challenges. IEEE Trans. Neural Syst. Rehabil. Eng..

[29] Scott N.R. (1967). Myoelectric control of prostheses and orthoses. Bull. Prosthet. Res..

[30] Luo J., Liu C., Yang C. (2019). Estimation of EMG-Based Force Using a Neural-Network-Based Approach. IEEE Access.

[31] Wimalasena L.N., Braun J.F., Keshtkaran M.R., Hofmann D., Gallego J.L., Alessandro C., Tresch M.C., Miller L.E., Pandarinath C. (2021). Estimating muscle activation from EMG using deep learning-based dynamical systems models. Cold Spring Harb. Lab..

[32] Geethanjali P. (2016). Myoelectric control of prosthetic hands: A state-of-the-art review. Med. Dev..

[33] Kamavuako E.N., Rosenvang J.C., Bøg M.F., Smidstrup A., Erkocevic E., Niemeier M.J., Jensen W., Farina D. (2013). Influence of the feature space on the estimation of hand grasping force from intramuscular EMG. Biomed. Signal Process. Control.

[34] Kuriki H.U., De Azevedo F.M., Takahashi L.S.O., Mello E.M., Filho R.D.F.N., Alves N. (2012). The Relationship between Electromyography and Muscle Force. EMG Methods for Evaluating Muscle and Nerve Function.

[35] Lee S.W., Kim J.H., Jun J., Ha J.W., Zhang B.T. Overcoming Catastrophic Forgetting by Incremental Moment Matching. Proceedings of the 31st Conference on Neural Information Processing Systems.

[36] Broderick T., Boyd N., Wibisono A., Wilson A.C., Jordan M.I. Streaming Variational Bayes. Proceedings of the Advances in Neural Information Processing Systems.

[37] Li Z., Hoiem D. Learning without forgetting. Proceedings of the European Conference on Computer Vision.

[38] Kirkpatrick J., Pascanu R., Rabinowitz N., Veness J., Desjardins G., Rusu A.A., Milan K., Quan J., Ramalho T., Barwinska A.G. (2017). Overcoming catastrophic forgetting in neural networks. Proc. Natl. Acad. Sci. USA.

[39] Fu Y., Chai T. (2007). Nonlinear multivariable adaptive control using multiple models and neural networks. Automatica.

[40] Elfelly N., Dieulot J.-Y., Benrejeb M., Borne P. (2010). A new approach for multimodel identification of complex systems based on both neural and fuzzy clustering algorithms. Eng. Appl. Artif. Intell..

[41] Elfelly N., Dieulot J.-Y., Borne P. (2008). Neural approach for the multimodel representation of complex processes. Int. J. Comput. Commun. Control.

[42] Xue Z.K., Li S.Y. (2006). Multimodel modelling and predictive control based on local model networks. Control Intell. Syst..

[43] Chihi I., Abdelkrim A., Benrejeb M. (2016). Multimodel approach to characterise human handwriting motion. Biol. Cybern..

[44] Adeniran A.A., El Ferik S. (2017). Modeling and Identification of Nonlinear Systems: A Review of the Multimodel Approach—Part 1. IEEE Trans. Syst. Man Cybern. Syst..

[45] Greblicki W. (1994). Nonparametric identification of Wiener systems by orthogonal series. IEEE Trans. Autom. Control.

[46] Voros J. (1995). Identification of Nonlinear Dynamic Systems Using Extended Hammerstein and Wiener Models. Control-Theory Adv. Technol..

[47] Kumar P., Potluri C., Sebastian A., Chiu S., Urfer A., Naidu D.S., Schoen M.P. (2010). An adaptive multi sensor data fusion with hybrid nonlinear ARX and Wiener–Hammerstein models for skeletal muscle force estimation. WSEAS Trans. Syst..

[48] Abbasi-Asl R., Khorsandi R., Farzampour S., Zahedi E. (2011). Estimation of Muscle Force with EMG Signals Using Hammerstein-Wiener Model. Biomed. IFMBE Proc..

[49] Eskinat E., Johnson S.H., Luyben W.L. (1991). Use of Hammerstein models in identification of nonlinear systems. AIChE J..

[50] Sebastian A., Kumar P., Schoen M.P. (2011). Modelling surface electromyogram dynamics using Hammerstein-Wiener models with comparison of IIR and spatial filtering techniques. Int. J. Circuits Syst. Signal Process..

[51] Zhu Y. (2002). Estimation of an N-L-N Hammerstein-Wiener Model. Automatica.

[52] Mete S., Ozer S., Zorlu H. System identification using Hammerstein model. Proceedings of the Signal Processing and Communications Applications Conference.

[53] Ozer S., Zorlu H., Mete S. (2016). System identification application using Hammerstein model. Indian Acad. Sci..

[54] Chihi I., Sidhom L., Trabelsi M. Nonlinear Hammerstein-Wiener model-based Fault Detection Approach for Cascaded H-Bridge Multilevel Inverters. Proceedings of the IEEE-GCC Conference & Exhibition (IEEE-GCC 2019).

[55] Wang Z., Georgakis C. (2019). Identification of Hammerstein-Weiner models for nonlinear MPC from infrequent measurements in batch processes. J. Process Control.

[56] Kamavuako E.N., Englehart K.B., Jensen W., Farina D. (2012). Simultaneous and Proportional Force Estimation in Multiple Degrees of Freedom from Intramuscular EMG. IEEE Trans. Biomed. Eng..

[57] Djigan V.I. (2006). Multichannel parallelisable sliding window RLS and fast RLS algorithms with linear constraints. Int. J. Adapt. Control Signal Process..

[58] Ding F., Ding J. (2010). Least-squares parameter estimation for systems with irregularly missing data. Int. J. Adapt. Control Signal Process..

[59] Narenda K.-S., Balakrishman J. (1997). Adaptive control using multiple models. IEEE Trans. Autom. Control.

[60] Pappas S.P., Leros A.K., Katsikas S.K. (2006). Joint order and parameter estimation of multivariate autoregressive models using multimodel partitioning theory. Digit. Signal Process..

